# Deregulation of small non-coding RNAs at the *DLK1-DIO3* imprinted locus predicts lung cancer patient outcome

**DOI:** 10.18632/oncotarget.13133

**Published:** 2016-11-05

**Authors:** Katey S.S. Enfield, Victor D. Martinez, Erin A. Marshall, Greg L. Stewart, Sonia H.Y. Kung, Jhon R. Enterina, Wan L. Lam

**Affiliations:** ^1^ Department of Integrative Oncology, British Columbia Cancer Research Centre, Vancouver, B.C. V5Z 1L3, Canada

**Keywords:** DLK1-DIO3, imprinted locus, lung cancer, microRNA, piwi-interacting RNA

## Abstract

Deregulation of the imprinted *DLK1*-*DIO3* locus at chromosome 14q32.1-14q32.31 has been associated with developmental and respiratory disorders, including cancer. In lung cancer, deregulation of imprinting at *DLK1*-*DIO3* was recently described in smokers. Deregulated expression of a microRNA (miRNA) cluster mapping to this locus was also associated with patient outcome, suggesting the importance of this locus to lung cancer disease phenotypes. The *DLK1*-*DIO3* locus is complex, and encodes several protein-coding genes, in addition to long and short non-coding RNAs. While the role of miRNAs is established, the biological importance of another relevant class of small RNAs, PIWI-interacting RNAs (piRNAs), has not been investigated. When somatically expressed, piRNAs regulate gene transcription through DNA methylation. Interestingly, their expression patterns have been observed to be altered in cancer and correlated with patient outcome. Here, we characterize the somatic expression of piRNAs encoded at *DLK1*-*DIO3* in two independent cohorts of lung adenocarcinoma and lung squamous cell carcinoma and investigate their associations with patient outcome. We find that the expression of piRNAs encoded at *DLK1-DIO3* enhances the prognostic potential of small non-coding RNAs specific to this locus in predicting patient outcome, further emphasizing the importance of regulation at this locus in lung cancer.

## INTRODUCTION

Genomic imprinting is the process by which the expression of an allele is silenced by methylation dependant on parental origin [1]. Aberrant methylation patterns at imprinted loci resulting in expression changes of encoded transcripts are common in the pathogenesis of many diseases, including cancer [2, 3]. In humans, anomalous imprinting at the *DLK1-DIO3* locus at 14q32.1-14q32.31 has been associated with respiratory insufficiency and reduced thorax development, amongst many other developmentally-related disorders [4].

**Figure 1:**
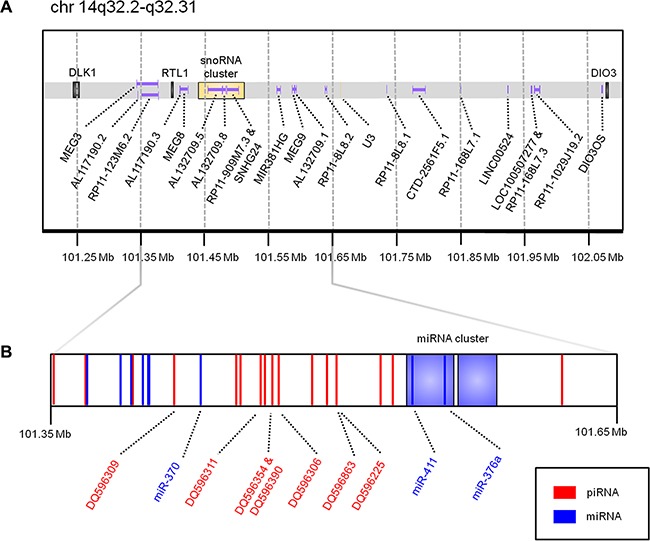
Schematic of the *DLK1*-*DIO3* imprinted locus Genomic coordinates are derived from the UCSC Genome Browser (hg19 build). (**A**) illustrates protein-coding genes (black), long non-coding RNAs (purple), and snoRNAs (yellow). (**B**) is a zoomed view of chr14:101,350,000-101,650,000 to highlight the genomic position of miRNAs previously associated with lung cancer patient outcome (*miR-370*, *miR-411*, and *miR-376a*), as well as the seven piRNAs identified as expressed in this study. miRNAs, including two large miRNA clusters containing 41 miRNAs in total, are coloured in blue; piRNAs are coloured in red.

The complexity of this locus is derived from the many protein-coding and non-coding RNAs it encodes. This locus encodes long non-coding RNAs (lncRNAs), and small non-coding RNAs (ncRNAs), including one of the largest microRNA (miRNA) clusters in the human genome (Figure 1A). Deregulation of small ncRNAs, mainly miRNAs, expressed from this locus has been associated with development and progression of different tumors, including lung, in both humans and mice [5–7]. While individual genes expressed from this locus have been associated with lung cancer patient outcome, a signature of three miRNAs has been shown to better predict overall survival and recurrence-free survival [8]. This combined prediction signature suggests that the analysis of multiple genes encoded at *DLK1-DIO3* may be more biologically informative than the analysis of any single gene.

**Figure 2:**
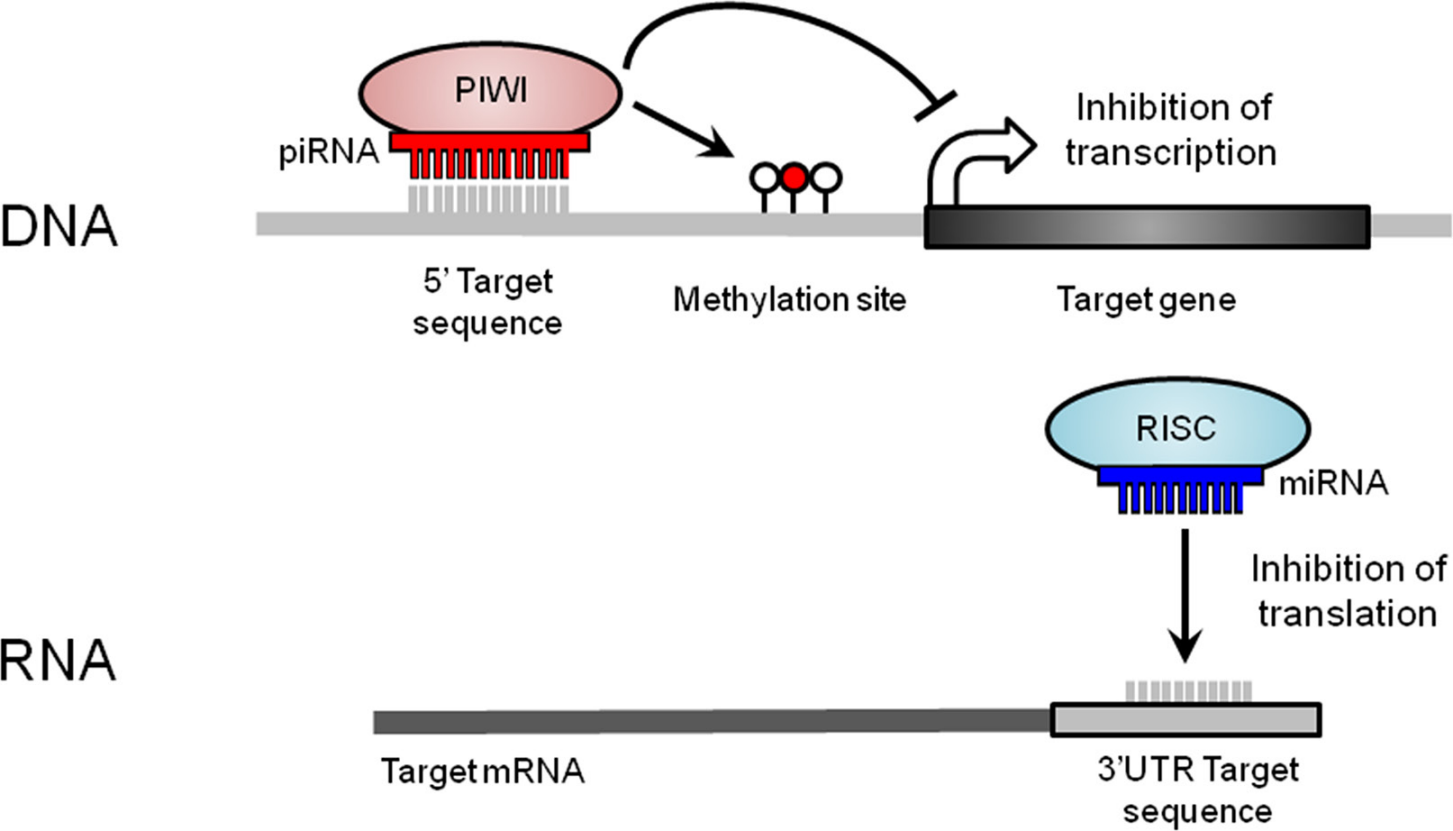
Small ncRNA-mediated mechanisms of gene expression regulation at the DNA and RNA levels At the DNA level, piRNAs form a complex with PIWI (P-element-induced wimpy testis) proteins. piRNAs first target DNA sequences through base complementarity. Then, the piRNA/PIWI complex recruits the silencing machinery required to induce new DNA methylation events (red lollipop) nearby the targeted region, repressing transcript expression. At the RNA level, miRNAs, together with a RNA-induced silencing complex (RISC), bind to a 3′ untranslated region (UTR) target sequence through base complementarity, which results in translational repression or mRNA degradation.

The role of other classes of small ncRNAs at this locus, such as PIWI-interacting RNAs (piRNAs), which act primarily as transcriptional regulators, has not yet been investigated in lung cancer (Figure 2). piRNAs have highly-conserved functions across species, including epigenetic silencing of transposable elements and regulation of imprinting in mice [9]. Although originally discovered in germ cells, recent evidence of their somatic expression in non-malignant human tissues and tumours suggests alternative functions and clinical importance [10–16].

In this study, we identify piRNAs expressed from *DLK1-DIO3* and determine whether their expression patterns enhance the prognostic value of small ncRNAs encoded at this clinically important locus. We have analyzed piRNA and miRNA expression profiles from two independent cohorts of non-small cell lung cancer (NSCLC) and investigated their relationship with patient outcome.

## RESULTS

### The *DLK1-DIO3* locus encodes somatically expressed piRNAs

**Figure 3:**
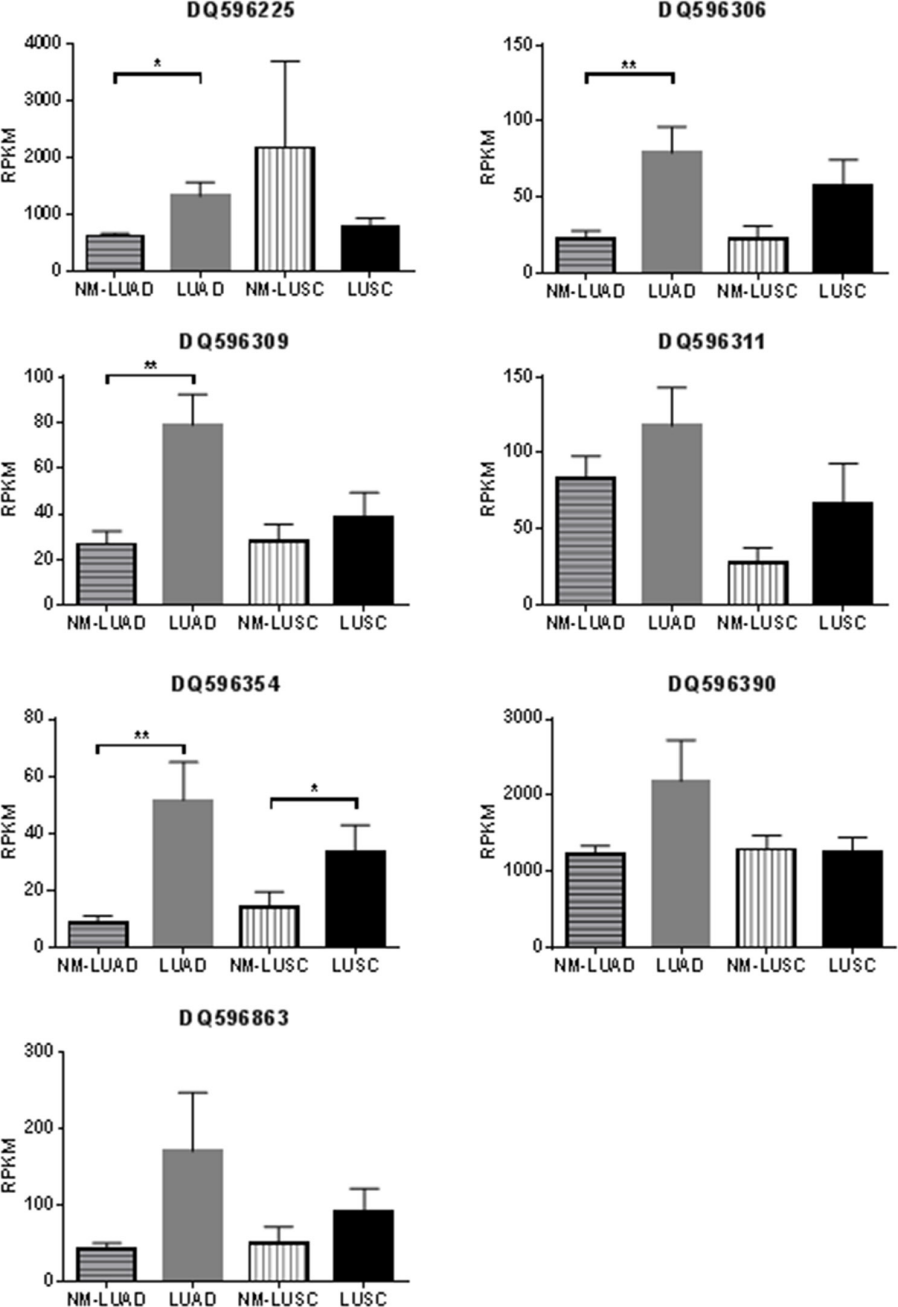
Histograms of piRNAs expressed in the discovery dataset (BCCA) Histograms display mean RPKM expression plus SEM in 84 paired non-malignant lung (NM-LUAD) and lung adenocarcinoma samples (LUAD), and 34 paired non-malignant lung (NM-LUSC) and lung squamous cell carcinoma (LUSC) samples. Significant *p*-values resulting from paired sign-rank analyses of gene expression are indicated as follows: *p < 0.05 **p < 0.01.

Deregulation of the *DLK1-DIO3* locus has been reported to be important to lung cancer biology, but the role of piRNAs derived from this locus has not yet been described. We analyzed expression data from two independent cohorts of lung adenocarcinoma (LUAD), lung squamous cell carcinoma (LUSC), and non-malignant lung samples to identify somatically-expressed piRNAs encoded at this locus (Table 1). Of the 138 piRNAs encoded at the *DLK1-DIO3* locus, seven were expressed in LUAD, LUSC, and non-malignant lung samples in the discovery cohort (DQ596225, DQ596306, DQ596309, DQ596311, DQ596354, DQ596390, DQ596863) (Figure 1B, Figure 3). Expression of all seven piRNAs was validated in the external cohort (Supplementary Figure S1). Interestingly, these somatically expressed piRNAs are encoded exclusively in the imprinted locus. In the discovery cohort of paired tumour and non-malignant lung tissues, four of seven somatically expressed piRNAs (DQ596225, DQ596306, DQ596309, DQ596354) were significantly overexpressed in LUAD and one piRNA (DQ596309) was overexpressed in LUSC (Figure 3). In the external dataset, two piRNAs (DQ596225, DQ596390) were validated to be significantly differentially expressed. Furthermore, six of seven piRNAs were significantly differentially expressed between LUAD and LUSC, with higher expression observed in LUSC (Supplementary Figure S1).

**Table 1:** Clinical features of lung cancer patient cohorts

Clinical Feature	Discovery Cohort *n* (%)		External Cohort *n* (%)	
Histological Subtype	LUAD	LUSC	LUAD	LUSC
Tumour	84	34	163	220
Non-malignant	84	34	46	45
Smoking History				
Current	35 (42)	11 (32)	44 (27)	57 (26)
Never	25 (30)	1 (3)	17 (10)	7 (3)
Former	20 (24)	22 (65)	98 (60)	147 (67)
Gender				
Male	24 (33)	10 (29)	86 (53)	165 (75)
Female	56 (67)	24 (71)	77 (47)	47 (21)
Age				
Range	45–90	58–88	40–86	39–84
Median	71	70	64	68
Stage				
IA	29 (35)	3 (9)	37 (23)	36 (16)
IB	19 (23)	11 (32)	51 (31)	61 (28)
IIA	13 (15)	4 (12)	13 (8)	30 (14)
IIB	5 (6)	7 (21)	23 (14)	40 (18)
IIIA	10 (12)	2 (6)	21 (13)	31 (14)
IIIB	0 (0)	1 (3)	6 (4)	10 (5)
IV	2 (2)	1 (3)	12 (7)	3 (1)

### A combined miRNA+piRNA signature better predicts overall survival of lung adenocarcinoma patients

**Figure 4:**
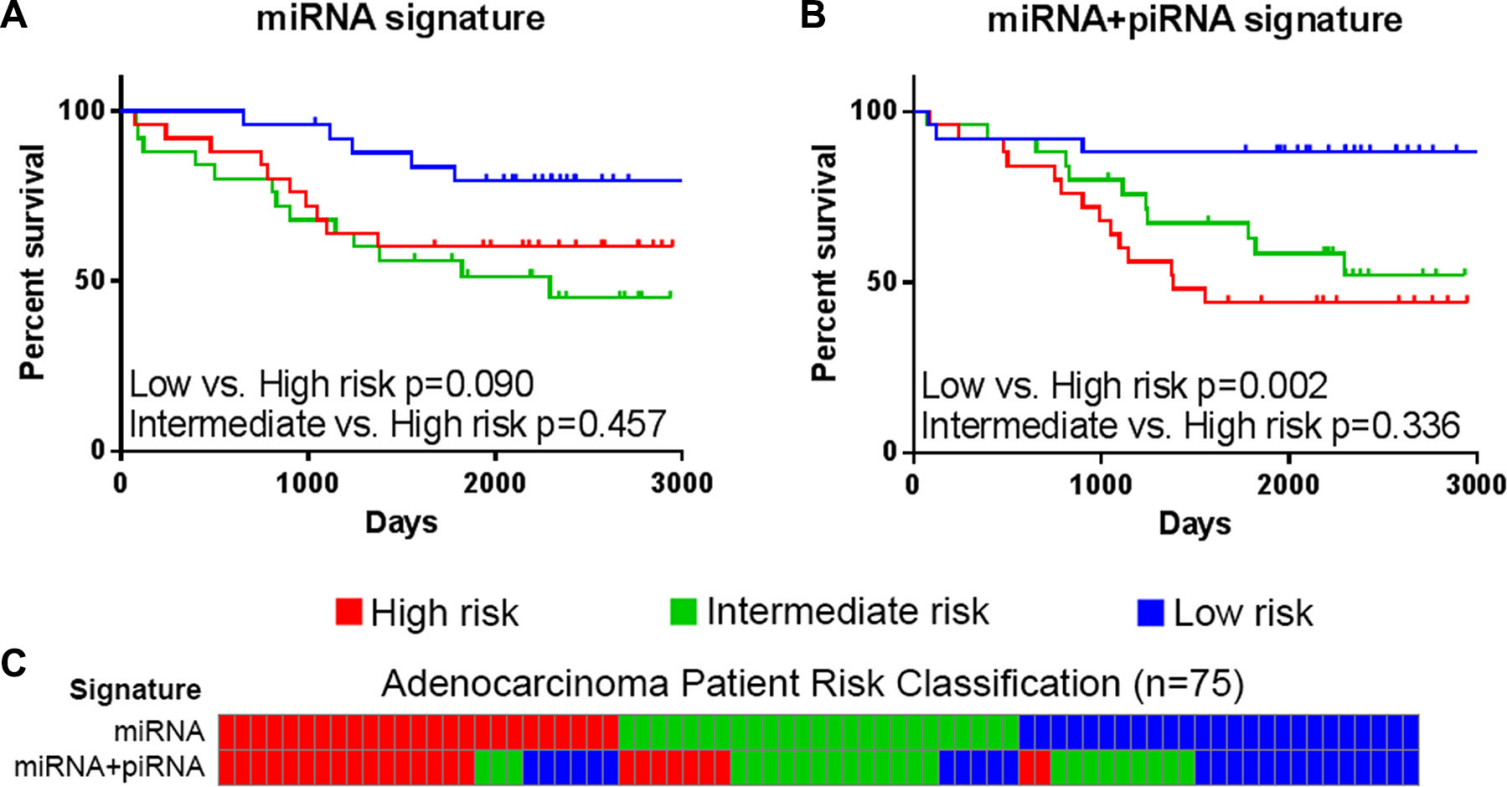
Overall survival of risk groups as defined in the lung adenocarcinoma discovery dataset (BCCA) (*n* = 75) Kaplan-Meier curves of high (red), intermediate (green), and low (blue) risk groups as defined by (**A**) the miRNA signature and (**B**) the miRNA+piRNA signature are shown. Log-rank *p*-values of select survival comparisons are shown. (**C**) Patients are ordered by their miRNA signature-based risk classification (top) in order to illustrate the re-classification that occurs when the miRNA+piRNA signature (bottom) is applied to the dataset.

Previous work has shown that a multi-miRNA classifier (*miR-370*, *miR-376a*, and *miR-411*) was able to predict LUAD patient outcome (Figure 1B) [8]. We applied this signature to our discovery dataset of LUAD and assessed the ability to predict patient overall survival (OS). Patient risk scores, indicating risk of death, were derived from a Cox proportional hazard model composed of these miRNAs. LUAD patients were divided into low, intermediate, and high risk groups and subjected to log-rank survival analysis. While this miRNA signature is able to classify low risk patients in the discovery dataset, the intermediate and high risk groups are not well segregated (Figure 4A). In the external dataset, the miRNA signature is better able to stratify LUAD patient risk groups and achieves marginal significance (low risk vs. high risk *p* = 0.051) (Supplementary Figure S2).

Next, we investigated if piRNAs expressed from the *DLK1-DIO3* locus could predict LUAD patient outcome. Just as the consideration of multiple miRNAs produced a predictive signature, we hypothesize the consideration of multiple piRNAs expressed from this locus could result in a similar signature. Interestingly, while piRNA expression alone was unable to significantly predict OS in univariate or multivariate analysis, the incorporation of piRNA expression into the miRNA signature improved the stratification of patients into risk groups. The final survival model was selected by adding different combinations of the seven expressed piRNAs to the miRNA Cox proportional hazard model, and the model with the lowest *p*-value was used to calculate patient risk scores. The final survival model included the three-miRNA signature and four piRNAs encoded at this locus (DQ596306, DQ596309, DQ596390, and DQ596863), and will be referred to as the miRNA+piRNA signature.

Approximately one-third of patients from each risk group are reclassified by the miRNA+piRNA signature (Figure 4C). Low risk LUAD patients had significantly improved outcome compared to both high (*p* = 0.002) and intermediate (*p* = 0.015) risk groups (Figure 4B). In the external cohort, high-risk LUAD patients had significantly worse outcome compared to both low (*p* = 0.037) and intermediate (*p* = 0.011) risk groups (Supplementary Figure S2). In the external dataset the Kaplan-Meier curves of the low and medium risk groups were overlapping; suggesting the miRNA+piRNA signature is better able to categorize intermediate risk patients into either high or low risk groups. When the new low and intermediate risk groups are combined in this dataset, the OS prediction improves (*p* = 0.004) (Figure 7, Supplementary Figure S2). A family-wise error rate (FWER) adjustment was applied to the *p*-values using the stringent Bonferroni method in order to test the robustness of the signature. Even after adjustment, the majority of the miRNA+piRNA signature *p*-values passed the new significance threshold (Figure 7).

### The miRNA+piRNA signature is able to predict overall survival of lung squamous cell carcinoma patients

**Figure 5:**
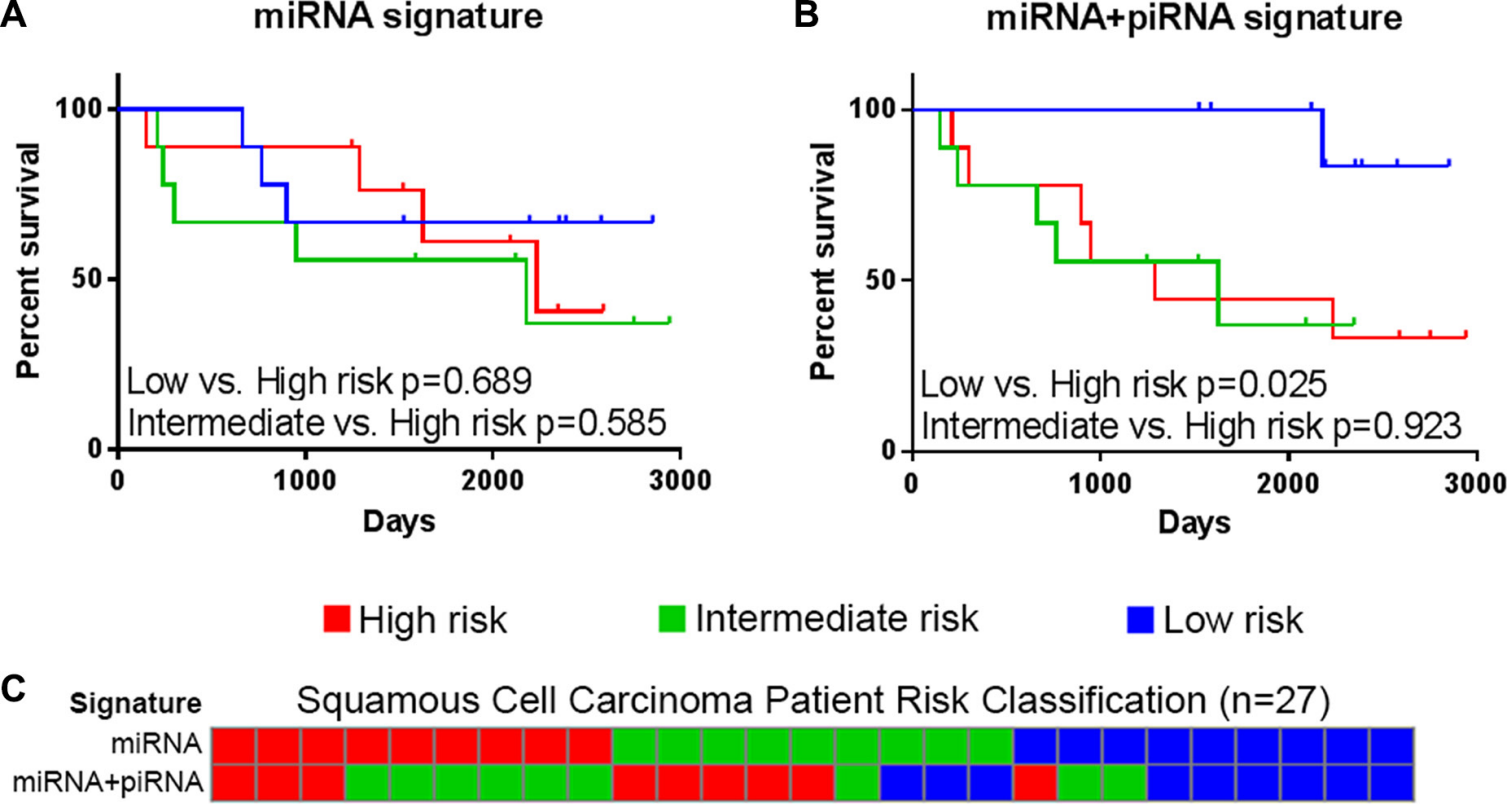
The miRNA+piRNA signature predicts overall survival in lung squamous cell carcinoma patients Risk scores calculated based on (**A**) miRNA signatures and (**B**) miRNA+piRNA signatures. Patients were assigned to high (red), intermediate (green), and low (blue) risk groups and Kaplan-Meier survival curves were compared. (**C**) Risk group classifications were compared based on the miRNA-only (top) and miRNA+piRNA (bottom) signatures. Risk group colors are the same as in the above panels.

The previously-described miRNA signature has not been assessed in the other major subtype of NSCLC, LUSC. In both our discovery and external datasets, LUSC patient risk groups as defined by the miRNA signature did not have significantly different OS outcomes (Figure 5A). Similarly, the LUSC patient risk groups stratified by piRNA expression did not have significantly different OS. However, as was shown in LUAD, the miRNA+piRNA signature was also able to classify LUSC patients into risk groups with distinct OS outcomes in both the discovery (Figure 5B) and external datasets (Figure 7, Supplementary Figure S3). *P*-values remained significant after Bonferroni adjustment in the external dataset. All but one of the intermediate risk LUSC patients were reclassified into either high or low risk groups by the miRNA-piRNA signature in the discovery dataset (Figure 5C). Furthermore, the intermediate and high risk Kaplan-Meier curves overlap, again suggesting that the miRNA+piRNA signature may identify two risk groups rather than three in some cases.

### The miRNA+piRNA signature identifies patients at risk of recurrence-free survival

**Figure 6:**
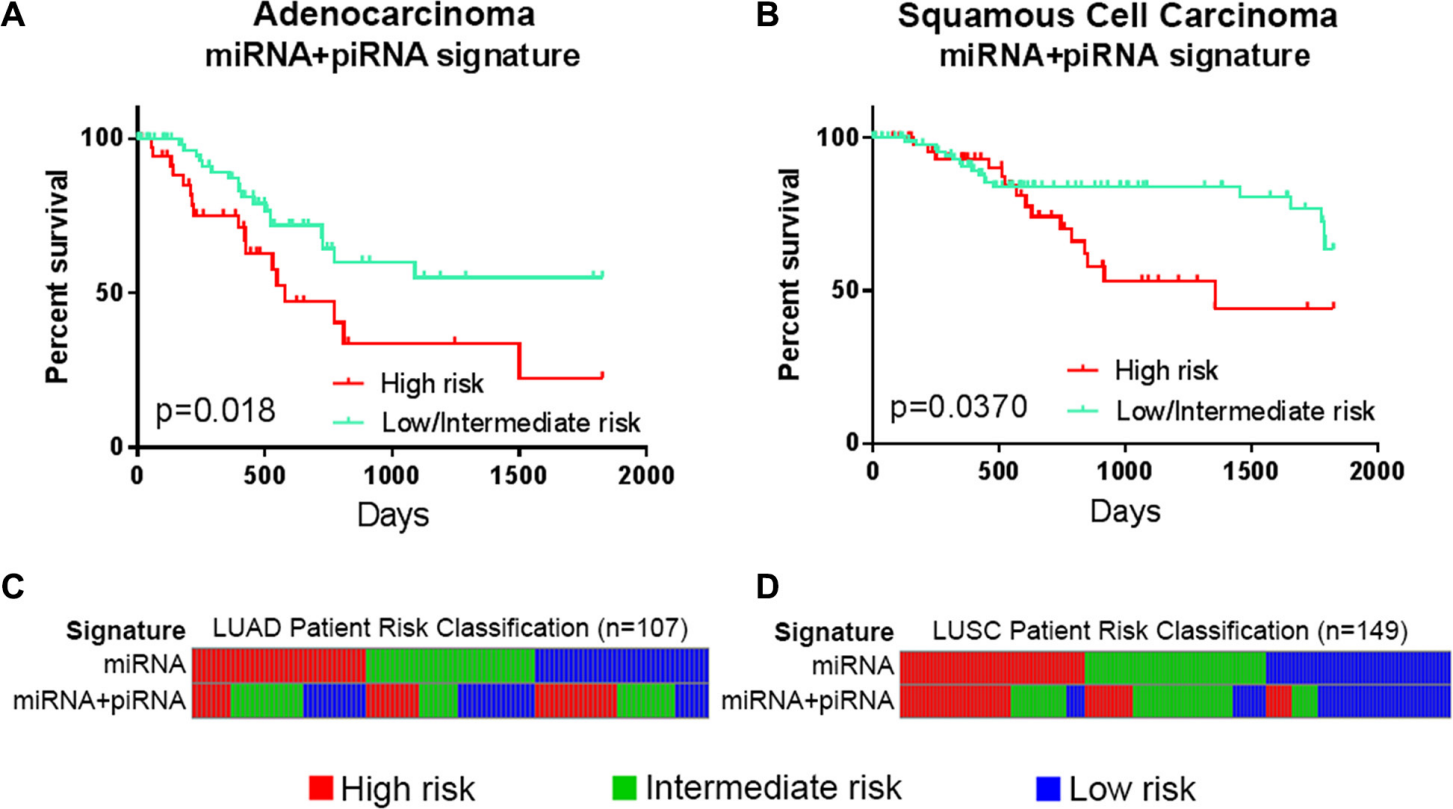
Performance of small ncRNA-based signatures predicting recurrence-free survival in non-small cell lung cancer Risk groups as defined in the external dataset of (**A**) lung adenocarcinoma (LUAD, *n* = 107) and (**B**) lung squamous cell carcinoma (LUSC, *n* = 149). Kaplan-Meier curves of high risk groups (red) compared to the combined low and intermediate risk groups (turquoise) as defined by the miRNA+piRNA signature are shown. (**C**) LUAD patients and (**D**) LUSC patients are ordered by their miRNA signature-based risk classification (top) in order to illustrate the re-classification that occurs when the miRNA+piRNA signature (bottom) is applied to the dataset. Intermediate and low risk patients are represented by green and blue bars, respectively.

In the external dataset, we compared RFS data of risk groups defined by the miRNA signature, the piRNA signature, and the miRNA+piRNA signature. Only the miRNA+piRNA signature was able to stratify two patient risk groups with statistically different outcomes. Similarly to OS, RFS classifications by the miRNA+piRNA signature were statistically significant in both LUAD (*p* = 0.018) and LUSC histological subtypes (*p* = 0.037) (Figure 6), but did not pass Bonferroni adjustment (Supplementary Figure S4).

**Figure 7:**
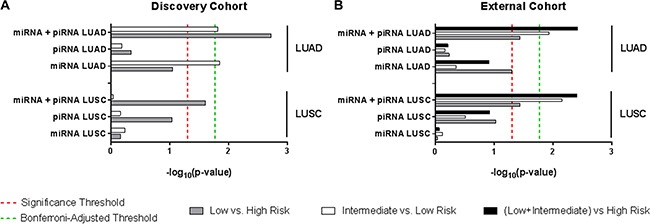
Log-rank *p*-value summary for overall survival predictions Bar lengths represent the –log_10_(*p*-value) of each signature for LUAD (top) and LUSC (bottom) patients from (**A**) the discovery cohort and (**B**) the external cohort. Comparison across different risk groups are as follows: low vs. high risk (grey bars), intermediate vs. low risk (white bars), low vs. intermediate risk (black bars). Significance thresholds are established at *p*-value = 0.05 (red dashed line indicates –log_10_ 0.05), and at *p*-value = 0.017 (Bonferroni-adjusted *p*-value) (green dashed line indicates –log_10_ 0.017).

## DISCUSSION

Here, we establish that piRNAs are expressed at the *DLK1-DIO3* locus, and suggest their relevance to lung cancer prognostics. We demonstrate the biological importance of multiple small ncRNA species through associations with NSCLC patient outcome. Incorporating both piRNA and miRNA expression in the classification of LUAD patients into risk groups improves classification compared to either small RNA species alone. In addition, stratification considering piRNA expression broadens the applicability of the signature to LUSC, which was not possible with miRNA expression alone. These findings highlight the complexity of the *DLK1-DIO3* locus and underscore its clinical relevance to both major histological subtypes of NSCLC.

The enhanced prediction of patient outcome may be linked to the additional level of regulation of gene expression provided by piRNAs, as well as the specific features of the seven piRNA expressed from the *DLK1-DIO3* locus. In order to regulate repetitive elements, single piRNAs are often encoded at multiple loci throughout the genome. However, piRNAs encoded at one locus are thought to function by regulating DNA methylation in target regions thereby acting as regulators of gene expression [17, 18]. We identify seven somatically expressed piRNAs solely encoded at this locus, suggesting these piRNAs may function to regulate methylation of target genes. Malignancy-associated methylation changes at this locus were recently described in lung cancer [19]; therefore, it is possible these piRNAs are involved in the deregulation of methylation patterns of this locus during lung tumourigenesis. Further studies will be required to determine whether deregulation of methylation at the *DLK1-DIO3* locus is mediated by piRNAs or by alternative mechanisms.

Although the function of somatically-expressed piRNAs has not yet been fully established, mounting evidence indicates they may serve as prognostic markers in a variety of tumor types, including gastric (RFS), colon (RFS), breast (lymph node positivity), kidney (OS) and head and neck (OS) cancer [11, 13–16, 20, 21]. Moreover, piRNAs, as other small ncRNAs, are stable in biofluids and formalin-fixed paraffin-embedded material, highlighting the potential of piRNA-based prognostic markers across a variety of tumour types.

In summary, our results provide further evidence of the involvement of imprinting-regulated small ncRNAs into NSCLC biology. The tissue-specific expression patterns of piRNAs and other small ncRNAs warrants further studies in order to establish their role across a wider spectrum of tissue types.

## MATERIALS AND METHODS

### Clinical cohorts and sample purity estimates

A description of discovery and external datasets and their clinical features can be found in Table 1.

### Discovery cohort

Paired tumour and non-malignant samples were obtained from the British Columbia Cancer Agency (BCCA) under Research Ethics Board approval. Tumour sections were microdissected to achieve > 80% cell purity, as directed by a pathologist [21, 22].

### External cohort

In The Cancer Genome Atlas (TCGA) dataset, purity estimates for tumour samples were publically available (http://cancergenome.nih.gov/). A purity cut-off of > 70% was applied according to previously published studies [23]. This was to make expression profiles more comparable between datasets, and to reduce contaminating sequences derived from alternative cell types since piRNA and miRNA expression is highly tissue specific.

### RNA extraction and small RNA sequencing

For our discovery cohort (BCCA), total RNA was extracted using Trizol reagent (Thermofisher, Waltham, MA, USA), according to the manufacturer's instructions, and eluted in RNase-free water. RNA concentration and quality was determined using a NanoDrop™ 2100 spectrophotometer, and samples were stored at −80°C. Sequencing analysis protocol was performed in the same manner for both the discovery and external (TCGA) cohorts [24]. Small RNA sequencing libraries were generated at Canada's Michael Smith Genome Sciences Centre and sequenced using Illumina HiSeq2000 instruments. For miRNA expression levels in our discovery cohort, reads were aligned using the Burrows-Wheeler Aligner (Version 0.5.7) and quantified against a miRNA annotation reference (miRBase Mature microRNAs Version 20). miRNA expression levels for the external cohort (TCGA) were accessed and retrieved in January 2015 using the TCGA data portal.

piRNA expression was determined as previously described [13]. Briefly, reads were first subject to quality control to exclude non-biological artifacts. Then, unaligned reads (in FASTQ format) were trimmed by size (retained reads ≥ 23 bp) and quality score (Phred quality scores ≥ 20) in order to enrich for high-quality reads mapping to piRNAs. Using the PartekFlow™ platform (Partek Inc., MO, USA), high-quality reads were mapped to the human genome (GRCh37/hg19) using the Spliced Transcripts Alignment to a Reference (STAR) aligner [25]. Reads were quantified by an Expectation/Maximization (E/M) algorithm [26] using a piRNA-specific annotation file generated from the piRNABank database (http://pirnabank.ibab.ac.in/) [27]. Partek Genome Suite (PGS) was used to further process and filter quantified files. Reads per kilobase of exon model per million mapped reads (RPKM) was used to scale and normalize read count [28].

### Small non-coding RNA differential expression analysis

Small ncRNAs were considered expressed if they had a scaled/normalized expression value ≥ 1 in at least 10% of both the discovery and external datasets. In the discovery cohort, small ncRNA expression from the paired tumour and non-malignant lung samples were compared by the sign-rank test, and between histological subtypes by the Mann Whitney *U*-test. In the external cohort, all two-group comparisons were performed using the Mann Whitney *U*-test. Significance threshold was established at *p*-value ≤ 0.05.

### Survival analysis

Univariate analysis: Cases were grouped based on piRNA expression tertiles, and survival analysis was conducted by log-rank test. For piRNAs with expression of 0 RPKM in > 1/3 of samples, cases were dichotomized into those with RPKM = 0 and those with RPKM > 0.

Cox proportional hazard model: Samples that had complete miRNA expression (RPKM), piRNA expression (RPKM), and survival data (overall survival or recurrence-free survival) were considered for Cox proportional hazard models. In addition to miRNAs previously associated with lung cancer patient outcome (*miR-370*, *miR-376a*, *miR-411*), Cox proportional hazard models including combinations of the seven expressed piRNAs, and combinations of the miRNAs and piRNAs were analyzed. The model with the lowest *p*-value was chosen for further analysis. Patient risk scores were generated per model by multiplying the expression value of a given gene by its hazard coefficient, and then summing the transformed gene expression values per sample [21]. Risk scores were ranked and divided into tertiles of high, intermediate, and low risk. Risk group Kaplan-Meier survival curves were then compared using the log-rank method. Significance threshold was established at *p*-value ≤ 0.05. Raw *p*-values were then adjusted using the Bonferroni method, resulting in an adjusted *p*-value cut-off of ≤ 0.017.

## SUPPLEMENTARY MATERIALS


