# Supplementary material for: Deregulation of small non-coding RNAs at the DLK1-DIO3 imprinted locus predicts lung cancer patient outcome
Source: Oncotarget. 2016 Nov 5;7(49):80957–66. doi: 10.18632/oncotarget.13133 (PMC5348368; doi:10.18632/oncotarget.13133)
Supplement: Supplementary file 1 [file oncotarget-07-80957-s001.pdf]

Deregulation of small non-coding RNAs at the *DLK1-DIO3* imprinted locus predicts lung cancer patient outcome

Supplementary Materials

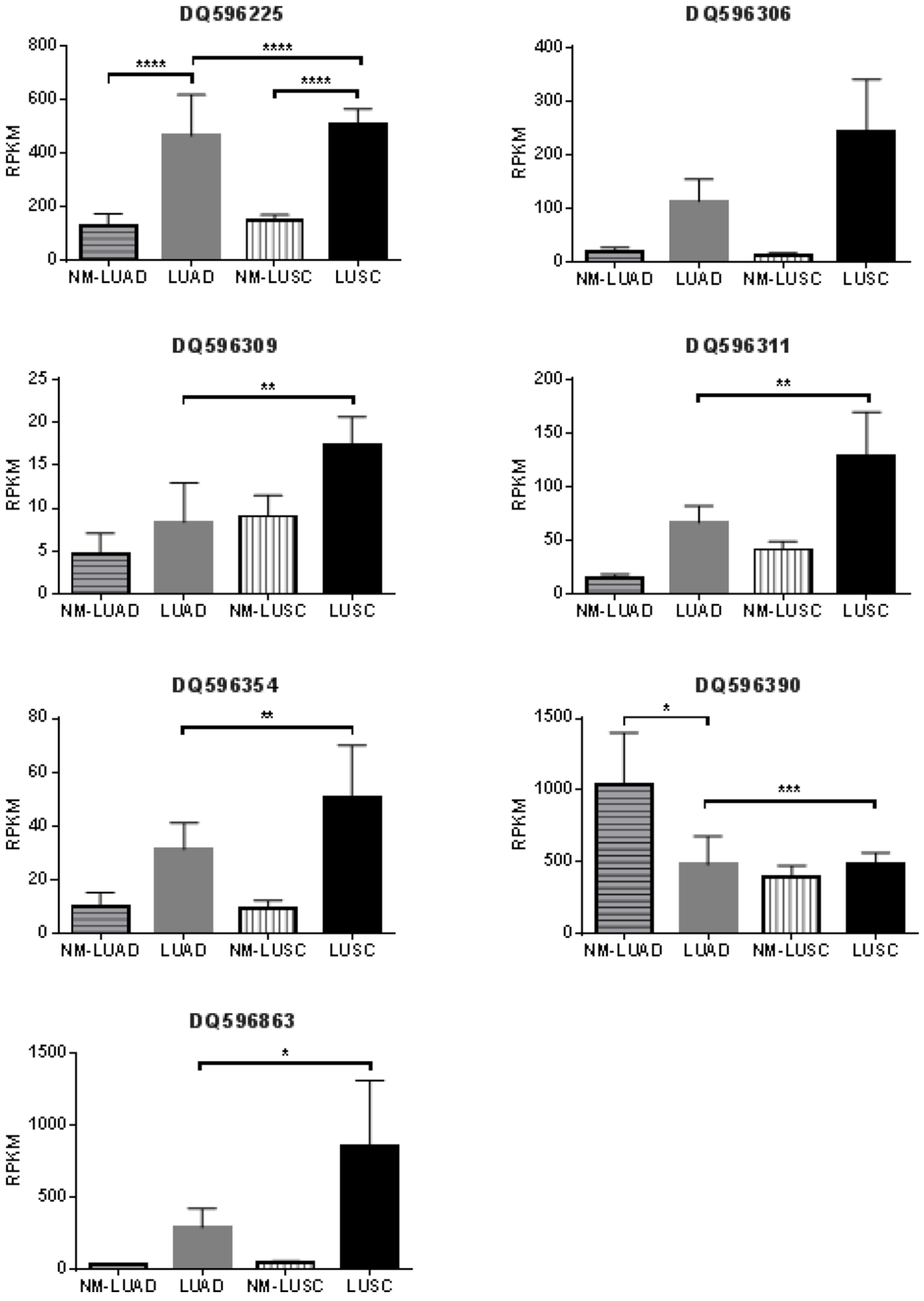

Supplementary Figure S1: Histograms of piRNAs expressed in the external dataset (TCGA). Histograms display mean RPKM expression plus SEM in 46 non-malignant lung (NM-LUAD) and 163 lung adenocarcinoma samples (LUAD), and 45 non-malignant lung (NM-LUSC) and 220 lung squamous cell carcinoma (LUSC) samples. Significant p-values resulting from Mann-Whitney *U* tests comparing gene expression are indicated as follows: $*p < 0.05$ $**p < 0.01$. $***p < 0.001$ $****P < 0.0001$.

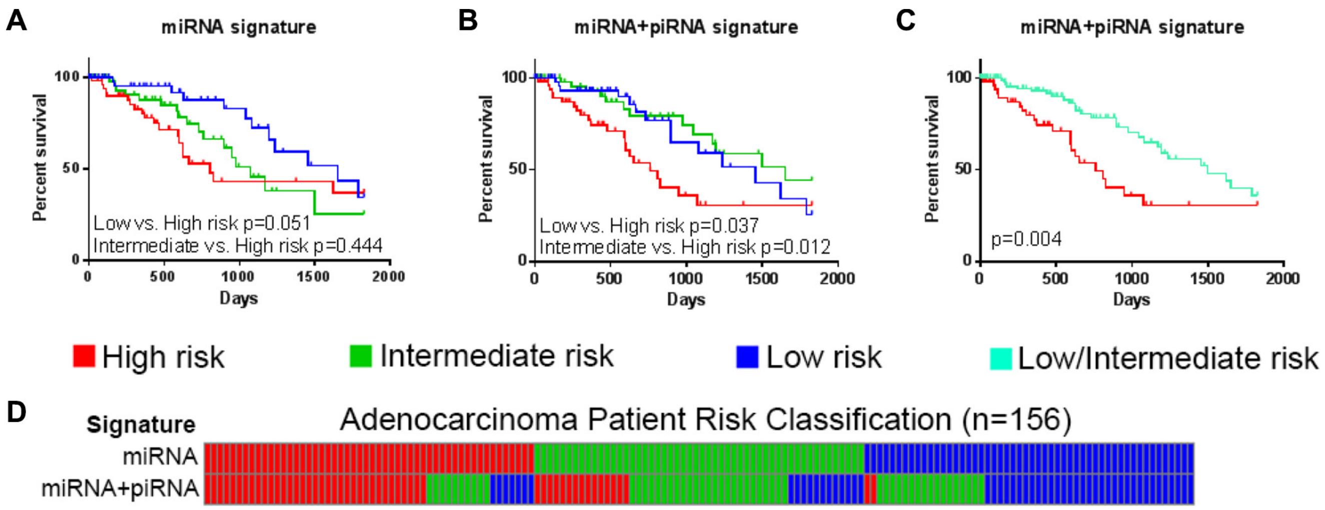

Supplementary Figure S2: Overall survival of risk groups as defined in the lung adenocarcinoma external dataset (TCGA) (n = 156). Kaplan-Meier curves of high (red), intermediate (green), and low (blue) risk groups as defined by (**A**) the miRNA signature and (**B**) the miRNA+piRNA signature are shown. Panel (**C**) combines the low and intermediate risk groups (turquoise) and compares survival to the high risk group. Log-rank p-values of select survival comparisons are shown. (**D**) Patients are ordered by their miRNA signature-based risk classification (top) in order to illustrate the re-classification that occurs when the miRNA+piRNA signature (bottom) is applied to the dataset.

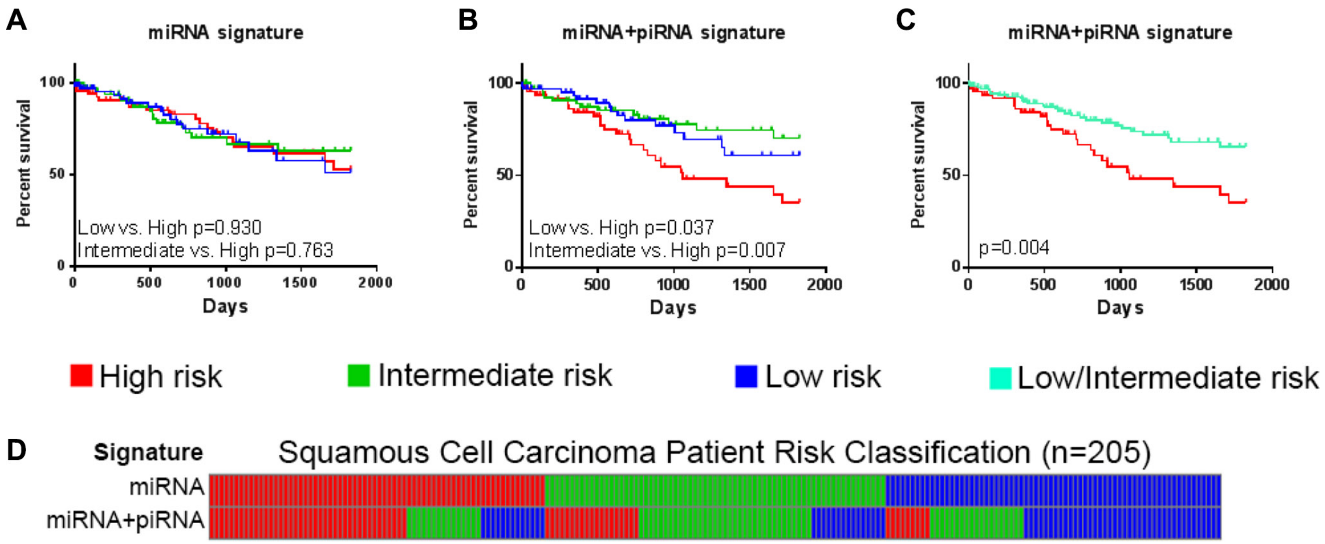

Supplementary Figure S3: Overall survival of risk groups as defined in the lung squamous cell carcinoma external dataset (TCGA) (n = 205). Kaplan-Meier curves of high (red), intermediate (green), and low (blue) risk groups as defined by (**A**) the miRNA signature and (**B**) the miRNA+piRNA signature are shown. Panel (**C**) combines the low and intermediate risk groups (turquoise) and compares survival to the high risk group. Log-rank p-values of select survival comparisons are shown. (**D**) Patients are ordered by their miRNA signature-based risk classification (top) in order to illustrate the re-classification that occurs when the miRNA+piRNA signature (bottom) is applied to the dataset.

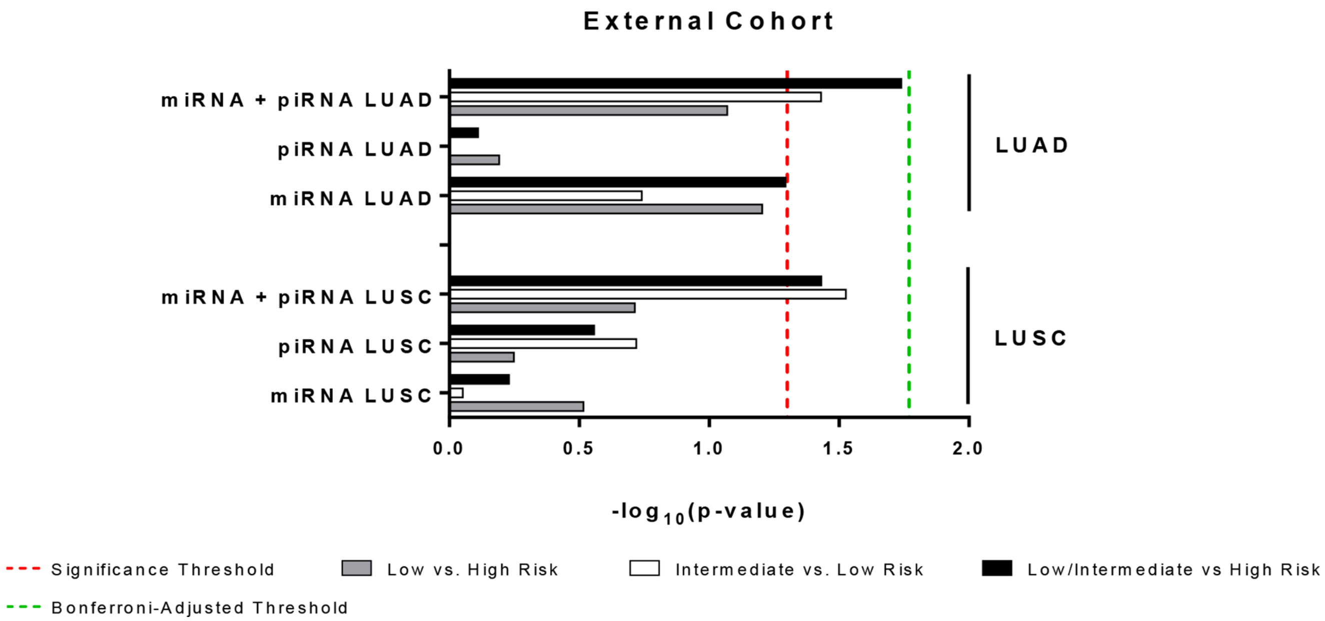

Supplementary Figure S4: Log-rank *p*-value summary for recurrence-free survival predictions in the external dataset. Bar lengths represent the $-\log_{10}$(*p*-value) of each signature for LUAD (top) and LUSC (bottom) patients from the discovery cohort. Comparison across different risk groups are as follows: low vs. high risk (grey bars), intermediate vs. low risk (white bars) low vs. intermediate risk (black bars). Significance threshold is established at *p*-value = 0.05 (red dashed line indicates $-\log_{10}$ 0.05), and at *p*-value = 0.017 (Bonferroni-adjusted *p*-value) (green dashed line indicates $-\log_{10}$ 0.017).
